# QTLs for Seed Vigor-Related Traits Identified in Maize Seeds Germinated under Artificial Aging Conditions

**DOI:** 10.1371/journal.pone.0092535

**Published:** 2014-03-20

**Authors:** Zanping Han, Lixia Ku, Zhenzhen Zhang, Jun Zhang, ShuLei Guo, Haiying Liu, Ruifang Zhao, Zhenzhen Ren, Liangkun Zhang, Huihui Su, Lei Dong, Yanhui Chen

**Affiliations:** 1 College of Agronomy, Synergetic Innovation Center of Henan Grain Crops and National Key Laboratory of wheat and Maize Crop Science, Henan Agricultural University, Zhengzhou, China; 2 College of Agronomy, Henan University of Science and Technology, Luoyang, China; China Agricultural University, China

## Abstract

High seed vigor is important for agricultural production due to the associated potential for increased growth and productivity. However, a better understanding of the underlying molecular mechanisms is required because the genetic basis for seed vigor remains unknown. We used single-nucleotide polymorphism (SNP) markers to map quantitative trait loci (QTLs) for four seed vigor traits in two connected recombinant inbred line (RIL) maize populations under four treatment conditions during seed germination. Sixty-five QTLs distributed between the two populations were identified and a meta-analysis was used to integrate genetic maps. Sixty-one initially identified QTLs were integrated into 18 meta-QTLs (mQTLs). Initial QTLs with contribution to phenotypic variation values of R^2^>10% were integrated into mQTLs. Twenty-three candidate genes for association with seed vigor traits coincided with 13 mQTLs. The candidate genes had functions in the glycolytic pathway and in protein metabolism. QTLs with major effects (R^2^>10%) were identified under at least one treatment condition for mQTL2, mQTL3-2, and mQTL3-4. Candidate genes included a calcium-dependent protein kinase gene (302810918) involved in signal transduction that mapped in the mQTL3-2 interval associated with germination energy (GE) and germination percentage (GP), and an hsp20/alpha crystallin family protein gene (At5g51440) that mapped in the mQTL3-4 interval associated with GE and GP. Two initial QTLs with a major effect under at least two treatment conditions were identified for mQTL5-2. A cucumisin-like Ser protease gene (At5g67360) mapped in the mQTL5-2 interval associated with GP. The chromosome regions for mQTL2, mQTL3-2, mQTL3-4, and mQTL5-2 may be hot spots for QTLs related to seed vigor traits. The mQTLs and candidate genes identified in this study provide valuable information for the identification of additional quantitative trait genes.

## Introduction

Seed vigor, an important and complex agronomic trait, is controlled by multiple factors such as genetic and physical purity, mechanical damage, and physiological conditions [1]–[3]. Seeds with high vigor can exhibit high germination rates, resistance to environmental stress, and high crop yields [4], [5]. Moreover, high-quality seeds that ensure uniform germination and growth that lead to increased production are important to growers, and seed vigor depends fundamentally on the potential of the seed itself to grow under favorable growth conditions and under adverse stress conditions. The ability to predict seed vigor using an artificial aging test is indispensable for ensuring rapid and uniform emergence of plants and for maximizing potential productivity under a wide range of field conditions.

Sensitivity of seeds to artificial aging has been used successfully to rapidly evaluate and predict seed vigor. High vigor seeds germinate normally after being subjected to artificial aging treatments, but low vigor seeds produce abnormal seedlings or die. Several physiological and biochemical processes have been identified that occur during artificial aging of seeds. For example, oxidative damage to DNA and proteins is likely to be involved in seed aging [6], and the formation of sugar–protein adducts or isoaspartyl residues may be factors contributing to the loss of protein function during artificial aging [7], [8]. In contrast, antioxidants, heat shock proteins (HSPs), and enzymes that repair protein damage may be involved in ameliorating the effects of artificial aging on seed vigor [7], [9]–[11]. Stress-related proteins and enzymes may also play a role in seed vigor. Prieto-Dapena et al. [10] reported that seed-specific overexpression of the sunflower heat stress transcription factor HaHSFA9 in tobacco enhanced the accumulation of HSPs and improved resistance of seeds to artificial aging [12]. Mutations in the rice aldehyde dehydrogenase 7 (OsALDH7) gene resulted in seeds that were more sensitive to artificial aging conditions and accumulated more malondialdehyde than wild-type seeds, implying that this enzyme plays a role in maintaining seed viability by detoxifying the aldehydes generated by lipid peroxidation [13]. A high level of a membrane lipid-hydrolyzing phospholipase D (PLDa1) appeared to be detrimental to seed quality, but attenuation of PLDa1 expression improved oil stability, seed quality, and seed vigor [14]. Lipoxygenases (LOXs) have also been reported to be involved in seed deterioration [15]. Overaccumulation of protein-l-isoaspartate *O*-methyltransferase (PIMT1, encoding IAMT in *Arabidopsis*) reduced the accumulation of l-isoaspartyl residues in seed proteins and increased germination vigor. Conversely, reduced PIMT1 accumulation was associated with an increase in the accumulation of l-isoaspartyl residues in the proteome of freshly harvested dry mature seeds, resulting in heightened sensitivity to aging treatments and the loss of seed vigor under stressful germination conditions [7].

Although environmental conditions during seed formation, harvest, and germination are important, genetic factors also have substantial impacts on seed vigor [16]–[20]. Genetic loci associated with seed vigor have been identified in rice, barley, wheat, oilseed rape, and *Arabidopsis thaliana* using artificial aging tests [17], [18], [21]–[26]. In addition, proteome analyses of seed vigor in *A. thaliana* and maize revealed common features in seeds subjected to artificial aging [8], [11].

To our knowledge, only two reports on proteomic characterization of specific proteins associated with seed vigor have been published. The use of artificial aging treatments to map quantitative trait loci (QTLs) associated with seed vigor by linkage analysis in maize has not been reported. In this study, seed vigor experiments and QTL analyses using two recombinant inbred line (RIL) populations and molecular markers were conducted under controlled conditions of seed deterioration to (1) identify QTLs for traits related to seed vigor, (2) integrate the QTLs identified across the two RIL populations to verify true QTLs, and (3) integrate candidate gene analyses with seed vigor QTL mapping across the two populations to assess the roles of candidate genes in natural variations in seed vigor in maize.

## Materials and Methods

### Population Development

The maize inbred line Shen137 was crossed with two other maize inbred lines, Yu82 and Yu537A, to make two connected F_1_ crosses during spring 2005 in Zhengzhou, Henan, China. The two crosses, Yu82 × Shen137 and Yu537A × Shen137, were self-pollinated by the single seed descent method to produce 208 and 212 F_10_ RILs, respectively, designated as Population 1 (Pop. 1) and Population 2 (Pop. 2), which were used to identify QTLs for traits related to seed vigor. Yu82 and Yu537A were derived from a Chinese Stiff Stalk germplasm, a heterotic group used broadly in China, while Shen137 was derived from a Chinese non-Stiff Stalk germplasm, also a heterotic group widely used in China.

### Artificial Aging Treatment

We used an improved method of artificial aging that was proposed originally by Zeng et al. [27]. Sample seeds from the 208 and 212 F_10_ RILs and their parent lines were treated at 45°C and 90% relative humidity for 0, 2, 4, and 6 days (0 d, 2 d, 4 d, and 6 d, respectively) using a thermostatic moisture regulator. The 0 d treatment was used as a control.

### Germination Experiment and Seed Vigor-related Trait Evaluation

The germination experiment followed a randomized complete block design with three replications and was conducted at 28°C in a growth chamber in 2011. Fine sand with a diameter of 0.05–0.2 mm was used as a sprouting bed. The sand was heated at 120°C for 2 h in a high-handed sterilization pan containing a 16 × 8 array of 40-mm-diameter wells. Two seeds were placed on top of 3.5 cm of sand in each well and then covered with 1.5 cm of sand. Fifty seeds of the parental lines and each RIL were selected to ensure good sowing quality. The seeds were incubated in a growth chamber at 28°C, 65% relative humidity, and illumination conditions of 4000 lux and a 14/10 (day/night) photoperiod with three replications. The number of germinated seeds was counted daily. Germination rate data were collected over an 8-day period after sowing and were used for QTL analysis when obvious differences between the parental lines and the RILs were observed. After the 8-day germination period, five plants of each RIL were selected randomly and dried in an oven at 65°C for 48 h and then weighed to obtain root dry weight (RDW) and seedling dry weight (SDW). The germination percentage (GP) was calculated as
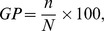
where *n* is the total number of germinated seeds and *N* is the total number of seeds tested. The germination energy (GE) was calculated as the number of germinated seeds on day 4 divided by the total number of seeds.

### Statistical Analysis of Phenotypic Data

Analysis of variance (ANOVA) was carried out to estimate genetic variation among the RILs for all of the traits. To normalize the variance, the germination percent was transformed to
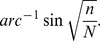



The frequency distribution of the traits was calculated to test the skewness of the traits toward the parents. Pearson correlation coefficients among the traits were computed using the statistical software package SPSS17.0 (SPSS Inc., Chicago, IL, USA) using the mean values of each trait.

### Construction of Molecular Marker Genetic Linkage Maps and QTL Analysis

Young-leaf samples were collected from seedlings of the two connected F_10_ RIL populations, and genomic DNA was extracted using the CTAB method [28]. Genotype data for single-nucleotide polymorphism (SNP) markers were analyzed using the Genome Studio Data analysis software (Illumina Inc., San Diego, CA, USA), which generates homozygous and heterozygous genotype clusters. In total, 3072 SNP markers were chosen to detect polymorphisms between the two parental lines, i.e., Yu82/Shen137 and Yu537A/Shen137.

Molecular genetic linkage maps were constructed with the maximum likelihood mapping function using JoinMap software version 4.0 [29]. Analysis of QTLs associated with seed vigor was conducted using composite interval mapping (CIM) with WinQTLcart 2.5 software [30]. The genome was scanned every 2 cM with a window size of 10 cM to exclude control markers around the tested interval [31]. Five control markers were identified by forward and backward regression. An empirical threshold level for declaring a QTL to be significant was an alpha level of 0.05 for each individual phenotypic trait across all traits. A maximum genetic distance of 50 cM was used to group all markers to chromosomes by performing 1000 random permutations. The phenotypic variation and additive effect explained by each QTL were estimated from the values expressed by the QTL peaks obtained from CIM.

For the additive effects of the QTLs, positive and negative values indicated that alleles from the normal maize inbreds Yu537A/Yu82 and the maize inbred Shen137, respectively, increased the trait scores. QTLs were named using the following nomenclature: “q” “+”artificial aging treatment days “+”trait abbreviation “+”population code “+”− “+”chromosome number “+”QTL number”.

### 1QTL Integration and Meta-analysis

To integrate QTL information for seed vigor-related traits obtained from the two connected RIL populations, the two molecular marker genetic linkage maps were integrated, and consensus QTLs were identified by meta-analysis [32]. The QTLs identified in the two connected RIL populations were projected onto the integrated map using their positions and confidence intervals shared by the two molecular marker genetic linkage maps. Ambiguous markers between the two molecular marker genetic linkage maps were deleted, which improved the accuracy of projection.

Meta-analysis was performed on the QTL clusters on each chromosome using BioMercator2.1 software [33]. The Akaike information criterion (AIC) was used to select the QTL model on each chromosome [34]. According to the AIC, the QTL model with the lowest AIC value is considered to be a significant model and indicates the number of meta-QTLs (mQTLs). A mQTL was declared only when it was shared by the two RIL populations. The number of mQTLs that gave the best fit to the results of a given linkage group was determined based on a modified AIC. mQTLs were named using the following nomenclature: “m”+“QTL”+“−” + “chromosome number” + “−” + “mQTL number.” QTL meta-analysis requires independent QTLs for the same trait to be obtained from different plant populations or different environmental conditions [35].

## Results

### Phenotypic Performance of Seed Vigor-related Traits in Maize

The Yu82, Yu537A, and Shen137 parental lines, designated P1, P2, and P3, respectively, exhibited clear differences in GE, GP, SDW, and RDW under the various treatment conditions. All four traits showed decreased values for the three parental lines after the 2 d, 4 d, and 6 d artificial aging treatments relative to the control (Table 1). Using GE as an example, the respective values of GE for P1, P2, and P3 were 0.92, 0.74, and 0.88 for the 0 d treatment (control); 0.80, 0.50, and 0.65 for the 2 d treatment; 0.80, 0.25, and 0.60 for the 4 d treatment; and 0.47, 0.12, and 0.33 for the 6 d treatment. The respective values for GE under the 2 d, 4 d, and 6 d aging treatments decreased relative to the controls by 0.12, 0.12, and 0.45 for Yu82; by 0.22, 0.49, and 0.62 for Yu537A; and by 0.23, 0.28, and 0.55 for Shen137.

**Table 1:** Phenotypic performance of traits related to seed vigor in three parental lines and two recombinant inbred lines (RILs) under normal and three artificial aging treatments.

	GE						GP		SDW (g)					RDW (g)			
	0 d	2 d	4 d	6 d	0 d	2 d	4 d	6 d	0 d		2 d	4 d	6 d	0 d	2 d	4 d	6 d
P1	0.92	0.80	0.80	0.47	1.00	0.90	0.69	0.30		0.53	0.50	0.42	0.30	0.32	0.23	0.25	0.21
P2	0.74	0.50	0.25	0.12	0.81	0.60	0.38	0.15		0.58	0.51	0.42	0.38	0.41	0.32	0.26	0.25
P3	0.88	0.65	0.60	0.33	0.97	0.85	0.51	0.27		0.47	0.38	0.39	0.33	0.29	0.24	0.23	0.21
P1 × P3 RIL																	
Mean ± S.D.	0.94±0.11	0.87±0.12	0.74±0.12	0.41±0.06	0.98±0.13	0.74±0.09	0.68±0.11	0.29±0.04		0.58±0.06	0.47±0.07	0.40±0.09	0.31±0.06	0.39±0.05	0.23±0.05	0.23±0.04	0.21±0.04
Range	0.75–0.98	0.83–0.96	0.57–0.88	0.35–0.58	0.84–1.00	0.70–0.89	0.47–0.77	0.25–0.37		0.56–0.71	0.32–0.64	0.25–0.54	0.24–0.57	0.33–0.48	0.17–0.31	0.22–0.48	0.18–0.37
Skewness	−1.25	−0.55	−0.31	−0.26	−0.86	−0.40	−0.07	0.10		0.34	0.05	−0.18	−0.30	0.31	−0.03	0.30	0.28
Kurtosis	0.89	0.41	−0.24	−0.27	0.61	−0.28	−0.06	−0.10		−0.14	0.03	0.17	−0.33	0.52	0.01	0.19	0.24
P2 × P3 RIL																	
Mean ± S.D.	0.89±0.08	0.59±0.11	0.40±0.07	0.25±0.04	0.87±0.08	0.77±0.15	0.52±0.08	0.24±0.04		0.53±0.11	0.43±0.11	0.40±0.07	0.36±0.05	0.37±0.06	0.28±0.05	0.25±0.04	0.23±0.04
Range	0.82–0.96	0.57–0.93	0.43–0.76	0.19–0.36	0.83–1.00	0.58–0.93	0.33–0.84	0.21–0.40		0.47–0.85	0.33–0.59	0.18–0.75	0.23–0.57	0.26–0.67	0.11–0.49	0.1–0.39	0.25–0.42
Skewness	−0.44	0.09	−0.05	−0.12	−0.98	0.14	0.06	−0.17		0.38	0.25	−0.12	0.14	0.61	0.14	0.08	0.17
Kurtosis	0.02	0.07	0.01	0.19	0.63	0.13	0.07	0.27		0.13	0.22	−0.11	0.16	0.89	0.16	0.04	0.18

P1, P2, and P3 indicate the Yu82, Yu537A, and Shen137 inbred lines, respectively. 0 d, control; 2 d, 2-day aging treatment; 4 d, 4-day aging treatment; 6 d, 6-day aging treatment; GE, germination energy; GP, germination percentage; RDW, root dry weight; SDW, seedling dry weight.

Similar trends for changes in GE, GP, SDW, and RDW in response to artificial aging treatments were observed in the RIL populations relative to the parents. The values of the four traits among the two RIL populations showed a pattern of continuous distribution around the mean (Table 1). The range of variability for the traits was large among the RILs and the traits differed substantially in response to the different aging treatments.

GE, GP, SDW, and RDW values showed significant positive correlations with each other in both of the RIL populations for the control and aging treatments except between SDW and GP, SDW and GE, RDW and GP, and RDW and GE for the control and 2 d aging treatments (Table 2). The correlation coefficients of all of the traits were higher for the three artificial aging treatments than for the 0 d control. In addition, phenotypic correlations among different treatments for the same seed vigor-related traits in the two RIL populations were significantly correlated (Table 3).

**Table 2:** Phenotypic correlations among four traits related to seed vigor for four treatments in two recombinant inbred line populations.

Treatment	Trait	GP	GE	SDW	RDW	Treatment	GP	GE	SDW	RDW
0 d	GP		0.52**	0.13	0.11	2 d		0.98**	0.02	0.04
	GE	0.78**		0.28	0.13		0.79**		0.02	0.05
	SDW	0.19*	0.20**		0.91**		0.21**	0.32**		0.92**
	RDW	0.22**	0.20**	0.21**			0.19*	0.28**	0.91**	
4 d	GP		0.96**	0.60**	0.61**	6 d		0.97**	0.44**	0.43**
	GE	0.94**		0.59**	0.60**		0.82**		0.44**	0.45**
	SDW	0.58**	0.63**		0.94**		0.66**	0.60**		0.93**
	RDW	0.84**	0.85**	0.82**			0.50**	0.46**	0.82**	

Correlation coefficients above the diagonal line in each quadrant of the table are for the Yu537 × Shen137 recombinant inbred line, and correlation coefficients below the diagonal line are for the Yu82 × Shen137 recombinant inbred line. 0 d, control; 2d, 2-day aging treatment; 4 d, 4-day aging treatment; 6 d, 6-day aging treatment; GE, germination energy; GP, germination percentage; RDW, root dry weight; SDW, seedling dry weight.

*Significant at P = 0.05, **Significant at P = 0.01.

**Table 3:** Phenotypic correlations among four treatments for four traits related seed vigor in two recombinant inbred line populations.

Trait	Treatment	0 d	2 d	4 d	6 d	Treatment	0 d	2 d	4 d	6 d
GE	0 d		0.73**	0.58**	0.45**	SDW		0.58**	0.30**	0.25**
	2 d	0.57**		0.58**	0.48**		0.31**		0.56**	0.43**
	4 d	0.55**	0.61**		0.69**		0.42**	0.39**		0.71**
	6 d	0.41**	0.41**	0.50**			0.25**	0.35**	0.47**	
GP	0 d		0.86**	0.84**	0.68**	RDW		0.86**	0.79**	0.65**
	2 d	0.70**		0.92**	0.76**		0.37**		0.91**	0.75**
	4 d	0.55**	0.66**		0.82**		0.39**	0.43**		0.82**
	6 d	0.46**	0.51**	0.64**			0.29**	0.35**	0.42**	

Correlation coefficients above the diagonal line in each quadrant of the table are for the Yu537 × Shen137 recombinant inbred line and correlation coefficients below the diagonal line are for the Yu82 × Shen137 recombinant inbred line. 0 d, control; 2 d, 2-day aging treatment; 4 d, 4-day aging treatment; 6 d, 6-day aging treatment; GE, germination energy; GP, germination percentage; RDW, root dry weight; SDW, seedling dry weight.

*Significant at P = 0.05, **Significant at P = 0.01.

### SNP Data Analysis and Genetic Linkage Map Construction

Of a total of 3072 SNP markers, 1397 and 1371 were polymorphic between the Yu82/Shen137 and Yu537A/Shen137 parents, respectively. The percentages of missing data in the genotyping of the mapping population across the 1397 and 1371 SNP loci were low at 1.32% and 1.68%, respectively. Statistical tests showed that most of the 2768 SNP markers followed the expected 1∶1 ratio. Ultimately, two genetic linkage maps consisting of all 10 maize chromosomes allocated to 10 linkage groups were constructed based on 1172 and 1139 SNP markers using Joinmap version 4.0 software [29]. The total lengths and average intervals were 1629.61 cM and 1.39 cM for Yu82/Shen137 and 1681.75 cM and 1.48 cM for Yu537A/Shen137. The 2768 SNP loci in the two linkage maps were consistent with the chromosome bin locations in the Maize Genetics and Genomics Database (MaizeGDB) maps.

### Identification of QTLs Associated with GE, GP, SDW, and RDW in the Two RIL Populations

Sixty-five QTLs for GE, GP, SDW, and RDW were mapped in seeds of the two RIL populations germinated under the control and artificial aging treatments, yielding 33 QTLs in Pop. 1 and 32 QTLs in Pop. 2 (Table 4). The QTLs mapped to all of the maize chromosomes except for chromosome 8. Individual QTLs explained from 5.40% to 11.92% of the phenotypic variation, while five of the QTLs accounted for more than 10% of the phenotypic variation.

**Table 4:** QTLs identified for four traits in seeds of the two RIL populations germinated under normal and three artificial aging conditions.

Trait	Treatment	QTL	Chr	Position (cM)	Marker Interval	LOD	R^2^ (%)	A
**Yu82 × Shen137**								
GE	N	***qnGE1-3***	3	84.53	PZE-103089927-PZE-103092676	3.05	5.61	−0.02
	2 d	***q2GE1-6***	6	85.82	SYN16940- PZE-106102131	2.71	5.91	0.03
	4 d	***q4GE1-1***	1	97.70	SYN29311- PZE-101152541	2.55	5.82	0.04
		***q4GE1-3***	3	56.40	PZE-103014908-PZE-103014908	3.69	10.67	−0.05
	6 d	***q6GE1-1***	1	115.86	PZE-101177728-PZE-101178540	2.89	6.54	0.04
GP	N	***qnGP1-3-1***	3	54.40	PZE-103014908-PZE-103014908	2.85	7.42	−0.02
		***qnGP1-3-2***	3	66.96	PZE-103032637-PZE-103036305	2.97	6.41	−0.02
		***qnGP1-5***	5	173.68	SYN14676-SYN33425	3.66	7.98	−0.02
	2 d	***q2GP1-3***	3	67.39	PZE-103036305-PZE-103033919	2.93	5.99	−0.03
		***q2GP1-5***	5	175.68	SYN33425-SYN33425	4.59	10.43	−0.04
		***q2GP1-6***	6	82.22	PZE-106097959-SYN16940	3.15	6.52	0.03
	4 d	***q4GP1-3-1***	3	52.40	SYN14585- PZE-103014908	2.76	6.67	−0.03
		***q4GP1-3-2***	3	67.39	PZE-103036305-PZE-103033919	2.93	5.99	−0.03
		***q4GP1-5-1***	5	166.97	SYN36222-SYN36222	2.74	6.81	−-0.03
		***q4GP1-5-2***	5	175.68	SYN33425-SYN33425	4.59	10.43	−0.04
		***q4GP1-6***	6	82.22	PZE-106097959-SYN16940	3.15	6.52	0.03
	6 d	***q6GP1-5***	5	173.68	SYN14676-SYN33425	2.92	6.47	−0.04
		***q6GP1-6***	6	39.06	PZE-106050123-PZE-106052536	2.61	5.74	0.04
SDW	N	***qnSDW1-5***	5	94.75	PZE-105102631–PZE-105101905	2.66	6.16	0.02
	2 d	***q2SDW1-4***	4	65.88	PZE-104018854–PZE-104018854	2.59	6.30	0.02
	4 d	***q4SDW1-4***	4	66.23	PZE-104018854–PZE-104021381	2.68	6.00	0.02
		***q4SDW1-6***	6	39.06	PZE-106050123–PZE-106052536	2.90	6.61	0.02
	6 d	***q6SDW1-4***	4	182.51	SYN24017–SYN16139	4.02	8.68	−0.03
		***q6SDW1-6***	6	92.93	PZE-106104150–PZE-106105801	2.53	5.40	0.02
RDW	N	***qnRDW1-1***	1	30.95	PZE-107094398–PZE-107094423	3.00	6.41	0.03
		***qnRDW1-10***	10	43.45	SYN18456–SYN18463	3.19	6.82	0.03
	2 d	***q2RDW1-7***	7	26.16	PZE-107017377–PZE-107057229	2.90	6.66	0.03
	4 d	***q4RDW1-1***	1	115.73	PZE-101177728–PZE-101178540	2.97	6.67	0.02
	6 d	***q6RDW1-1-1***	1	30.95	SYN13385–PZA03742.1	2.58	5.60	0.01
		***q6RDW1-1-2***	1	37.22	PZE-101064370–PZE-101071162	2.59	5.75	0.01
		***q6RDW1-4***	4	182.51	SYN24017–SYN16139	2.81	6.07	−0.01
		***q6RDW1-5***	5	73.42	PZE-105047885–PZE-105047805	3.63	7.88	−0.02
		***q6RDW1-6***	6	85.82	SYN16940–PZE-106102131	2.75	5.84	0.01
**Yu537A×Shen137**								
GE	N	***qnGE2-3***	3	156.45	PZE-103123325–SYN31220	2.63	6.54	0.02
		***qnGE2-4***	4	101.87	PUT-163a-31558543-1963– PZE-104084757	2.83	6.34	−0.02
		***qnGE2-5***	5	85.61	PZE-105102393–PZE-105109134	3.24	8.01	−0.02
	2 d	***q2GE2-9-1***	9	51.03	PZE-109047581–PZE-109046201	2.71	6.85	−0.03
		***q2GE2-9-2***	9	57.82	PZE-109055211–SYN34709	2.79	7.12	−0.03
	4 d	***q4GE2-3***	3	164.27	SYN30210–PZE-103118170	3.87	8.59	0.03
	6 d	***q6GE2-2***	2	159.02	PZE-102186216–SYN39029	2.93	6.12	−0.02
		***q6GE2-3-1***	3	60.54	PZE-103033919–PZE-103035540	3.03	6.30	0.02
		***q6GE2-3-2***	3	158.75	SYN31220–PZE-103118406	4.05	10.14	0.03
		***q6GE2-4***	4	173.25	PZE-104128483–PZE-104128458	3.06	6.30	−0.02
		***q6GE2-7***	7	18.80	SYN34669–PZE-107015480	3.73	8.05	−0.03
GP	N	***qnGP2-10***	10	16.54	PZE-110110920–SYN22564	2.52	5.97	−0.02
	2 d	***q2GP2-7***	7	67.33	PZE-107069959–SYN16900	2.52	7.34	0.02
	4 d	***q4GP2-7-1***	7	73.17	SYN16900–ZM013281-0173	3.01	8.08	0.03
		***q4GP2-7-2***	7	79.49	PZE-107094398–PZE-107097026	4.16	9.60	0.03
	6 d	***q6GP2-3-1***	3	60.54	PZE-103033919–PZE-103035540	2.64	5.92	0.02
		***q6GP2-3-2***	3	159.75	SYN31220–PZE-103118406	2.90	6.96	0.02
		***q6GP2-4***	4	74.10	PZE-104051877–PZE-104052411	2.77	5.81	−0.02
SDW	N	***qnSDW2-3***	3	111.57	SYN28063–PZE-103180642	2.52	5.39	−0.03
	2 d	***q2SDW2-2***	2	98.20	SYN8399- PZE-102122951	3.54	8.29	−0.03
		***q2SDW2-3***	3	126.76	PZE-103151399-SYN37386	3.03	6.29	−0.03
		***q2SDW2-10***	10	91.96	PZE-110038658–PZE-110036140	3.12	6.71	0.03
	4 d	***q4SDW2-2***	2	98.20	SYN8399– PZE-102122951	4.77	11.92	−0.04
		***q4SDW2-3***	3	126.76	PZE-103151399–SYN37386	3.28	6.92	−0.03
	6 d	***q6SDW2-2***	2	96.42	PZE-102118282–SYN33606	3.02	7.34	−0.03
RDW	N	***qnRDW2-3***	3	111.57	SYN28063–PZE-103180642	3.61	7.71	−0.03
		***qnRDW2-7***	7	71.17	SYN16900–PZE-107089819	2.72	5.67	−0.02
	2 d	***q2RDW2-2***	2	97.20	SYN8399– PZE-102122951	4.05	9.22	−0.03
		***q2RDW2-4***	4	88.56	PZE-104075457–PZE-104073719	3.32	7.02	−0.03
		***q2RDW2-10***	10	91.96	PZE-110038658–PZE-110036140	3.56	7.86	0.03
	4 d	***q4RDW2-2***	2	95.42	PZE-102118282–SYN33606	3.53	7.99	−0.02
	6 d	***q6RDW2-2***	2	97.20	SYN8399–PZE-102122951	3.46	7.86	−0.03

A, additive effect; GE, germination energy; GP, germination percentage; RDW, root dry weight; SDW, seedling dry weight.

### QTLs for GE

Four QTLs for GE were identified on chromosomes 3, 4, and 5 in the 0d control seeds of the two RIL populations with one QTL in Pop. 1 and three QTLs in Pop. 2 (Table 4). The contributions of the QTLs to phenotypic variation ranged from 5.61% to 8.01%. The three QTLs in Pop. 2 were responsible for 20.89% of the phenotypic variation. The positive alleles of *qnGE1-3*, *qnGE2-4*, and *q6GE2-5* were contributed by Shen137. For each RIL in the two populations, molecular markers positioned close to the peaks of the QTLs were categorized by genotypic class (i.e., either Yu82/Yu537A or Shen137), and their respective GE values for seeds germinated under the control treatment were averaged. The respective average GE values in the untreated RILs corresponding to the Shen137 and Yu82 alleles of markers associated with the QTLs were 0.85 and 0.79 for *qnGE1-3*, 0.83 and 0.79 for *qnGE2-4*, 0.84 and 0.80 for *qnGE2-5*, and 0.78 and 0.84 for *qnGE2-3*.

Three QTLs for GE were identified on chromosomes 6 and 9 in seeds subjected to the 2d treatment with one QTL in Pop. 1 and two QTLs in Pop. 2 (Table 4). The contributions of the QTLs to phenotypic variation ranged from 5.91% to 7.12%. The two QTLs in Pop. 2 were responsible for 13.97% of the phenotypic variation. The positive alleles of *q2GE1-6* in Pop. 1 were contributed by Yu82, while the positive alleles of *q2GE2-9-1* and *q2GE2-9-2* in Pop. 2 were contributed by Shen137. The respective average GE values corresponding to the Shen137 and Yu82 alleles in the 2d treatment RILs were 0.79 and 0.72 for *q2GE1-6*, 0.73 and 0.75 for *q2GE2-9-1*, and 0.72 and 0.76 for *q2GE2-9-2*.

Three QTLs for GE were identified on chromosomes 1 and 3 in seeds subjected to the 4d treatment with two QTLs in Pop. 1 and one QTL in Pop. 2 (Table 4). The contributions of the QTLs to phenotypic variation ranged from 5.82% to 10.67%. The two QTLs in Pop. 1 were responsible for 16.49% of the phenotypic variation. The positive alleles of *q4GE1-3* in Pop. 1 were contributed by Shen137. The respective average GE values corresponding to the Shen137 and Yu82 alleles in the 4d treatment RILs were 0.57 and 0.64 for *q4GE1-3*, 0.63 and 0.57 for *q4GE1-1*, and 0.58 and 0.52 for *q4GE2-3*.

Six QTLs for GE were identified on chromosomes 1, 2, 3, 4, and 7 in seeds subjected to the 6d treatment with one QTL in Pop. 1 and five QTLs in Pop. 2 (Table 4). The contributions of the QTLs to phenotypic variation ranged from 6.12% to 10.14%. The five QTLs in Pop. 2 were responsible for 36.91% of the phenotypic variation. The positive alleles of *q6GE2-2*, *q6GE2-4*, and *q6GE2-7* in Pop. 2 were contributed by Shen137. The respective average GE values corresponding to the Shen137 and Yu82 alleles in the 6d treatment RILs were 0.30 and 0.34 for *q6GE2-2*, 0.30 and 0.34 for *q6GE2-4*, 0.31 and 0.35 for *q6GE2-7*, 0.35 and 0.30 for *q6GE2-3-1*, 0.38 and 0.34 for *q6GE2-3-2*, and 0.31 and 0.36 for *q6GE1-1*.

### QTLs for GP

Four QTLs for GP were identified on chromosomes 3, 5, and 10 in the 0d control seeds of the two RIL populations with three QTLs in Pop. 1 and one QTL in Pop. 2 (Table 4). The contributions of the QTLs to phenotypic variation ranged from 5.97% to 7.98%. The alleles derived from Shen137 contributed to increased trait values. The respective average GP values corresponding to the Shen137 and Yu82 alleles of markers associated with the QTLs in the untreated RILs were 0.88 and 0.92 for *qnGP1-3-1*, 0.88 and 0.91 for *qnGP1-3-2*, 0.87 and 0.93 for *qnGP1-5*, and 0.86 and 0.88 for *qnGP2-10*.

Four QTLs for GP were identified on chromosomes 3, 5, 6, and 7 in seeds subjected to the 2d treatment with three QTLs in Pop. 1 and one QTL in Pop. 2 (Table 4). The contributions of the QTLs to phenotypic variation ranged from 5.99% to 10.47%. Shen137 alleles contributed to increased trait values for the *q2GP1-3* and *q2GP1-5* QTLs and Yu82/Yu537A alleles generally increased the trait values for the *q2GP1-6* and *q2GP2-7* QTLs. The respective average GP values corresponding to the Shen137 and Yu82 alleles in the 2d treatment RILs were 0.82 and 0.88 for *q2GP1-3*, 0.81 and 0.90 for *q2GP1-5*, 0.89 and 0.81 for *q2GP1-6*, and 0.84 and 0.81 for *q2GP2-7*.

Seven QTLs for GP were identified on chromosomes 3, 5, 6, and 7 in seeds subjected to the 4d treatment with five QTLs in Pop. 1 and two QTLs in Pop. 2 (Table 4). The contributions of the QTLs to phenotypic variation ranged from 5.99% to 10.47%. Yu82/Yu537A alleles contributed to increased trait values for the *q4GP1-6*, *q4GP2-7-1*, and *q4GP2-7-2* QTLs and Shen137 alleles generally increased the trait values for the other four QTLs. The respective average GP values corresponding to the Shen137 and Yu82 alleles in the 4d treatment RILs were 0.71 and 0.65 for *q4GP1-6*, 0.69 and 0.65 for *q4GP2-7-1*, 0.70 and 0.64 for *q4GP2-7-2*, 0.65 and 0.73 for *q4GP1-3-1*, 0.66 and 0.72 for *q4GP1-3-2*, 0.65 and 0.71 for *q4GP1-5-1*, and 0.66 and 0.72 for *q4GP1-5-2*.

Five QTLs for GP were identified on chromosomes 3, 4, 5, and 6 in seeds subjected to the 6d treatment with two QTLs in Pop. 1 and three QTLs in Pop. 2 (Table 4). The contributions of the QTLs to phenotypic variation ranged from 5.74% to 6.96%. Shen137 alleles contributed to increased trait values for the *q6GP1-5* and *q6GP2-4* QTLs and Yu82/Yu537A alleles generally increased the trait values for the three additional QTLs. The respective average GP values corresponding to the Shen137 and Yu82 alleles in the 6d treatment RILs were 0.40 and 0.49 for *q6GP1-5*, 0.41 and 0.46 for *q6GP2-4*, 0.48 and 0.40 for *q6GP1-6*, 0.46 and 0.41 for *q6GP2-3-1*, and 0.45 and 0.39 for *q6GP2-3-2*.

### QTLS for SDW

Two QTLs for SDW were identified on chromosomes 3 and 5 in the 0d control seeds of the two RIL populations with one QTL in Pop. 1 and one QTL in Pop. 2 (Table 4). The two QTLs accounted for 6.16% and 5.39% of the phenotypic variation, respectively. The positive allele of *qnSDW2-3* was contributed by Shen137. The respective average SDW values corresponding to the Shen137 and Yu82 alleles of markers associated with the QTLs in the untreated RILs were 0.49 and 0.45 for *qnSDW1-5* and 0.50 and 0.56 for *qnSDW2-3*.

Four QTLs for SDW were identified on chromosomes 2, 3, 4, and 10 in seeds subjected to the 2d treatment with one QTL in Pop. 1 and three QTLs in Pop. 2 (Table 4). The contributions of the QTLs to phenotypic variation ranged from 6.29% to 8.29%. The positive alleles of *q2SDW2-2* and *q2SDW2-3* were contributed by Shen137. The respective average SDW values corresponding to the Shen137 and Yu82 alleles of markers associated with the QTLs in the 2d treatment RILs were 0.43 and 0.49 for *q2SDW2-2*, 0.43 and 0.48 for *q2SDW2-3*, 0.88 and 0.85 for *q2SDW1-4*, and 0.48 and 0.43 for *q2SDW2-10*.

Four QTLs for SDW were identified on chromosomes 2, 3, 4, and 6 in seeds subjected to the 4d treatment with two QTLs in Pop. 1 and two QTLs in Pop. 2 (Table 4). The contributions of the QTLs to phenotypic variation ranged from 6.00% to 11.92%. The positive alleles of *q4SDW2-2* and *q4SDW2-3* were contributed by Shen137. The respective average SDW values corresponding to the Shen137 and Yu82 alleles of markers associated with the QTLs in the 4d treatment RILs were 0.37 and 0.44 for *q4SDW2-2*, 0.38 and 0.43 for *q4SDW2-3*, 0.40 and 0.36 for *q4SDW1-4*, and 0.39 and 0.35 for *q2SDW1-6*.

Three QTLs for SDW were identified on chromosomes 2, 4, and 6 in seeds subjected to the 6d treatment with two QTLs in Pop. 1 and one QTL in Pop. 2 (Table 4). The contributions of the QTLs to phenotypic variation ranged from 5.40% to 8.68%. The positive alleles of *q6SDW1-4* and *q6SDW2-2* were contributed by Shen137. The respective average SDW values corresponding to the Shen137 and Yu82 alleles of markers associated with the QTLs in the 6d treatment RILs were 0.29 and 0.24 for *q6SDW1-6*, 0.24 and 0.29 for *q6SDW1-4*, and 0.29 and 0.35 for *q6SDW2-2*.

### QTLS for RDW

Four QTLs for RDW were identified on chromosomes 1, 3, 7, and 10 in the 0d control seeds of the two RIL populations with two QTLs in Pop. 1 and two QTLs in Pop. 2 (Table 4). The contributions of the QTLs to phenotypic variation ranged from 5.67% to 7.71%. The positive alleles of *qnRDW2-3* and *qnRDW2-7* in Pop. 2 were contributed by Shen137. The respective average RDW values corresponding to the Shen137 and Yu82 alleles of markers associated with the QTLs in the untreated RILs were 0.45 and 0.42 for *qnRDW1-1*, 0.44 and 0.42 for *qnRDW1-10*, 0.33 and 0.40 for *qnRDW2-3*, and 0.34 and 0.39 for *qnRDW2-7*.

Four QTLs for RDW were identified on chromosomes 2, 4, 7, and 10 in seeds subjected to the 2d treatment with one QTL in Pop. 1 and three QTLs in Pop. 2 (Table 4). The contributions of the QTLs to phenotypic variation ranged from 6.67% to 9.22%. The positive alleles of *q2RDW2-2* and *q2RDW2-4* in Pop. 2 were contributed by Shen137. The respective average RDW values corresponding to the Shen137 and Yu82 alleles of markers associated with the QTLs in the 2d treatment RILs were 0.28 and 0.33 for *q2RDW2-2*, 0.28 and 0.33 for *q2RDW2-4*, 0.49 and 0.45 for *q2RDW1-7*, and 0.33 and 0.28 for *q2RDW2-10*.

Two QTLs for RDW were identified on chromosomes 1 and 2 in seeds subjected to the 4d treatment with one QTL in Pop. 1 and one QTL in Pop. 2 contributing 6.67% and 7.99%, respectively, to the phenotypic variation (Table 4). The positive allele of *q4RDW2-2* in Pop. 2 was contributed by Shen137. The respective average RDW values corresponding to the Shen137 and Yu82 alleles of markers associated with the QTLs in the 4d treatment RILs were 0.22 and 0.29 for *q4RDW2-2* and 0.19 and 0.15 for *q4RDW1-1*.

Six QTLs for RDW were identified on chromosomes 1, 2, 4, 5, and 6 in seeds subjected to the 4d treatment with five QTLs in Pop. 1 and one QTL in Pop. 2 (Table 4). The contributions of the QTLs to phenotypic variation ranged from 5.60% to 7.88%. The positive alleles of *q6RDW1-4* and *q6RDW1-5* in Pop. 1 and *q6RDW2-2* in Pop. 2 were contributed by Shen137. The respective average RDW values corresponding to the Shen137 and Yu82 alleles of markers associated with the QTLs in the 6d treatment RILs were 0.13 and 0.11 for *q6RDW1-1-1*, 0.13 and 0.11 for *q6RDW1-1-2*, 0.13 and 0.11 for *q6RDW1-6*, 0.10 and 0.14 for *q6RDW1-4*, 0.10 and 0.13 for *q6RDW1-5*, and 0.16 and 0.21 for *q6RDW2-2*.

### mQTL Analysis

The integrated genetic map for the two populations contained 1712 SNP markers and was 1712.6 cM long with an average of 1.00 cM between markers (Table 5). Meta-analysis was performed to identify mQTLs that were associated with the observed variation in the seed vigor traits. Eighteen mQTLs were identified from the 65 identified initially based on variation in the four seed vigor traits (Table 6). Sixty-one of the initial QTLs (93.85%) were integrated into the mQTL regions.

**Table 5:** Distribution of SNP markers on the integrated molecular genetic linkage map.

Chromosome	Number ofSNP markers	Marker intervalrange (cM)	Total geneticdistance (cM)	Average distance(cM)	Number ofintegrated QTLs
1	215	0–11.4	230.8	1.07	6
2	196	0–12.0	192.5	0.98	7
3	117	0–19.5	184.2	1.57	17
4	235	0–9.1	182.3	0.78	9
5	175	0–13.9	167.3	0.96	7
6	178	0–8.1	154.5	0.87	7
7	148	0–9.2	165.0	1.11	6
8	192	0–11.6	143.1	0.75	0
9	137	0–10.7	148.9	1.09	2
10	119	0–12.8	144.0	1.21	4
Total	1712		1712.6	1.00	65

**Table 6:** mQTLs for four traits in seeds of two connected RIL populations germinated under normal and three artificial aging conditions.

mQTL	Chr	Position(cM)	Confidenceinterval (cM)	Flankingmarker	Physicalinterval (b)	No. ofQTLs	Integrated QTLs	Candidategenes
mQTL1-1	1	36.23	23.93–48.60	PZE-101043682–PZE-101065758	29889332–48812512	3	*qnRDW1-1*,*q6RDW1-1-1*,*q6RDW1-1-2*	226532762
mQTL1-2	1	119.25	106.28–132.21	SYN24128–SYN34477	179459862–200303983	3	*q4GE1-1*,*q4RDW1-1*,*q6GE1-1*	224061823
mQTL2	2	99.72	94.50–104.93	PZE-102119932–SYN34721	160591358–174854687	6	q2SDW2-2, *q4SDW2-2*,*q6SDW2-2*, *q2RDW2-2*,*q4RDW2-2*, *q6RDW2-2*	
mQTL3-1	3	68.59	64.50–72.69	SYN36772–SYN37724	13312517–25865416	3	*qnGE1-3*, *q6GP2-3-1*,*q6GE2-3-1*	242056533,195605946,162459222
mQTL3-2	3	47.12	43.08–51.16	PZE-103052783–PZE-103075978	58972898–126109305	6	*q2GP1-3-1*, *q2GP1-3-2*, *q4GE1-3*, *q4GP1-3-1*, *q4GP1-3-2*, *qnGP1-3-2*	327195227,302810918,At1g45050
mQTL3-3	3	120.19	111.30–129.01	SYN37386–SYN28063	194157875–207200950	4	*qnSDW2-3*, *qnRDW2-3*,*q2SDW2-3*, *q4SDW2-3*	224143836,162459414,At5g19550
mQTL3-4	3	157.66	149.74–165.58	PZE-103115618–SYN20493	175554472–184720973	4	*qnGE2-3*, *q4GE2-3*,*q6GP2-3-2*, *q6GE2-3-2*	AT5G51440
mQTL4-1	4	57.61	45.20–70.02	PZE-104050909–PZE-104045752	68235181–79841743	3	*q2SDW1-4*, *q4SDW1-4*,*q6GP2-4*	
mQTL4-2	4	94.31	82.20–106.42	PZE-104066884–PZE-104087575	132214044–162274823	2	*qnGE2-4*, *q2RDW2-4*	195658029,226508796,At3g04120
mQTL4-3	4	186.52	175.22–197.82	SYN18852–PZE-104157368	225732497–240245730	3	*q6RDW1-4*, *q6GE2-4*,*q6SDW1-4*	326509331, AT1G57720
mQTL5-1	5	76.35	66.14–86.56	PZE-105075207–PZE-105111462	82955415–168450026	2	*qnGE2-5*, *qnSDW1-5*	
mQTL5-2	5	140.93	136.83–145.02	SYN36222–PZE-105179864	211582817–214427178	5	*qnGP1-5*, *q4GP1-5-2*,*q4GP1-5-1*, *q6GP1-5*	At5g67360
mQTL6-1	6	64.22	44.53–83.92	PZE-106038001–SYN38352	86257528–123864232	2	*q4SDW1-6*,*q6GP1-6*	45238345,At1g70730,AT1G09640
mQTL6-2	6	113.98	106.40–121.56	PZE-106083335–PZE-106105801	140906714–156368157	5	*q6SDW1-6*, *q6RDW1-6*,*q2GP1-6*, *q2GE1-6*,*q4GP1-6*	226247007
mQTL7-1	7	31.68	20.78–42.58	PZE-107013546–PZE-107032657	9916088–43707645	2	*q2RDW1-7*, *q6GE2-7*	*AT1G56340*
mQTL7-2	7	75.02	65.14–84.90	PZE-107069628–PZE-107096067	126410451–151965220	4	*qnRDW2-7*, *q4GP2-7-2*,*q4GP2-7-1*, *q2GP2-7*	AT3G21720
mQTL10-2	10	94.70	86.87–102.53	PZE-110021391–PZE-110043433	28838744–82745414	2	*q2RDW2-10*,*q2SDW2-10*	

The 18 mQTLs mapped to all chromosomes except for chromosomes 8 and 9 (Table 6). On average, each mQTL included 3.39 QTLs with a range of two to six QTLs for one to four traits. The initial QTLs included in mQTL5-2 were all detected based on one trait (GP) in the 0 d control seeds and in seeds subjected to the three artificial aging treatments. The QTLs in mQTL3-2, mQTL3-3, and mQTL3-4 were identified based on one trait under three treatment conditions; the QTLs in mQTL2 were identified based on two traits under two conditions; the QTLs in mQTL1-1, mQTL1-2, mQTL3-1, mQTL6-2, and mQTL7-2 were identified based one trait under two conditions; and the QTLs in the remaining mQTLs were identified based on two traits under one condition. Note that the initial QTLs with values of R^2^>10% were integrated into four mQTLs: mQTL2, mQTL3-2, mQTL3-4, and mQTL5-2.

## Discussion

Seed vigor depends on the physiological and genetic potential of the seeds and on the effects of environmental stress conditions that they are exposed to during germination. For maize, plant stand establishment and performance are strongly influenced by seed vigor [36], [37]. Producing high-quality seeds to stabilize crop yield poses a challenge for crop breeders, and a key to meeting this challenge is the elucidation of the molecular mechanisms underlying seed vigor traits. Little research has been published to date on the use of artificial aging treatments to clarify the molecular mechanisms associated with seed vigor traits in maize. In this study, two sets of connected RIL populations were evaluated for four seed vigor traits after seeds were subjected to three artificial aging treatments during germination.

### Phenotypic Variation among Parent Inbred Lines and their RILs after Different Aging Treatments

Because germination is affected by seed vigor, many researchers have used the germination rate as an indicator of seed vigor [17], [38], [39]. To predict the ability of seeds to perform under a wide range of field conditions, an artificial aging test was used in this study. The results showed that GE and GP decreased gradually with increasing duration of the artificial aging treatment and that the germination rates differed between the parental lines and the RILs with artificial aging (Table 1). In addition, correlations between the results from the untreated controls and the seeds subjected to artificial aging treatments were significant (Table 3), indicating that the artificial aging test provided a valid assessment of seed vigor.

### Genotypic Differentiation in Two Populations Reflects Adaptation to Artificial Aging Conditions

The selection of maize lines for adaption to climatic conditions has resulted in differences in seed vigor in various inbred lines. These differences in vigor are reflected by differences in responses to artificial aging conditions. Only one common QTL identified in this study was located near the chromosome region associated with GE at bin 3.02, but multiple population-specific QTLs were identified even though the two populations shared Shen137 as a common parent. Specifically, five QTLs on chromosomes 1, 3 (2), 5, and 6 that were contributed by the parental line Yu82 were identified in Pop. 1 and five QTLs on chromosomes 2, 3 (2), 4, and 7 that were contributed by the parental line Yu537A were linked in Pop. 2.

Among the 18 mQTLs, 9 mQTLs included initial QTLs for GE on chromosomes 1, 3, 4, 5, 6, and 7; 8 mQTLs included initial QTLs for GP on chromosomes 3, 4, 5, 6, and 7; 8 mQTLs included initial QTLs for SDW on chromosomes 2, 3, 4, 5, 6, and 10; and 10 mQTLs included initial QTLs for RDW on chromosomes 1, 2, 3, 4, 6, 7, and 10.

The *GP1-3* and *SDW2-3* QTLs identified in Pop. 1 and Pop. 2, respectively, were detected in seeds subjected to control, 2 d, and 4 d treatments, but not the 6 d treatment. The results suggested that the loci associated with seed vigor played an important role during the early stages of seed deterioration caused by artificial aging. The progressive decrease in germination vigor depends on the duration of artificial seed aging [40]. This may be due to the diverse negative effects of reduced protein synthesis on processes such as maintenance, repair, and normal resumption of metabolism and cell cycle activity, efficiency of detoxification, efficiency of signaling pathways, and/or the production and secretion of metabolites and plant hormones [40]. Oxidative stress has also been shown to be a causal factor in aging processes [40]. The extent of oxidative damage to nucleic acids, lipids, and proteins has been shown to increase with age [41].

In contrast, the *GP1-5* QTLs in Pop. 1 were all identified in seeds subjected to control, 2 d, 4 d, and 6 d treatments, with contributions to phenotypic variation of single QTLs ranging from 6.52% to 10.67%. These results indicated that the QTLs confer resistance to high-temperature and humidity conditions, which may deserve further study in marker-assisted selection (MAS). A cucumisin-like Ser protease gene (At5g67360) mapped to the *QTL1-5* interval. Increased protease activities were observed in potato seed-tubers, wheat seeds, and maize seeds during artificial aging, which resulted in a substantial reduction in soluble and storage proteins [11], [42], [43]. Notably, *SDW2-2* and *RDW2-2* QTLs in Pop. 2 were identified only with the 2 d, 4 d, and 6 d treatments, indicating that these loci strongly influence plant stand establishment after seed deterioration caused by high-temperature and humidity conditions.

### Synthesis of Initial QTLs across Two Populations Subjected to Different Artificial Aging Treatments

We examined the genetic correlations among traits by mQTL analysis. Co-localization of QTLs for the traits associated with seed vigor might indicate pleiotropy and/or tight linkage. In this study, 16 mQTLs (e.g., mQTL6-2, mQTL4-3, mQTL2, and mQTL3-2) included two to six initial QTLs for two to four of the seed vigor traits. These initial QTLs might indicate pleiotropy and/or tight linkage. For mQTL2, mQTL3-2, and mQTL3-4, an initial QTL with a major effect (R^2^>10%) was identified for at least one treatment condition, and corresponding candidate genes mapped to the mQTL intervals except for mQTL2. The chromosome regions for three mQTLs with high QTL co-localization might be hot spots of important QTLs for seed vigor traits. Validation of potential candidate genes from the three mQTLs would provide a reliable and feasible strategy for QTL cloning.

### Associations between QTLs and Candidate Genes in Maize

Two proteomics studies in maize [11], [44] and one study in *Arabidopsis* [39] identified 28, 40, and 83 differentially expressed proteins, respectively, that were related to artificial seed aging. We explored the association between mQTLs and these differentially expressed proteins in maize using a bioinformatics approach. Twenty-three differentially expressed proteins were located at bin loci of 13 mQTLs (Table 6). The proteins had functions related to responses to stress, molecular chaperones, hydrolase activity, energy, cell growth/division, protein targeting and storage, signal transduction, translation, protein metabolism, amino acid metabolism, and other processes. Basavarajappa et al. [45] suggested that seed aging affected metabolism and energy supply in maize, while Ching and Schoolcraft [46] showed that seed storage protein content decreased with seed aging. Various toxic compounds such as reactive oxygen species (ROS), aldehydes, and methylglyoxal can accumulate during seed aging, possibly resulting in reduced seed viability and decreased germination rates [44], [47]. Some proteins in aged maize seeds might interfere with signal transduction in responses to stress caused by heat shock, desiccation, peroxide, salt, or abscisic acid (ABA) [48], [49]. Rajjou et al. [39] showed that seed aging treatments strongly increased the extent of protein oxidation (carbonylation), which might cause loss of function for seed proteins and enzymes and/or enhance their susceptibility to proteolysis.

### The Glycolytic Pathway is Affected during Artificial Seed Aging

The functions of eight candidate genes located within the mQTLs identified in seeds subjected to artificial aging treatments were associated with the glycolytic pathway. The functions of two of the candidate genes were associated with stress responses. A stress-responsive glyoxalase family gene (226532762) mapped in the mQTL1-1 interval that was associated with RDW and an aldehyde dehydrogenase gene (45238345) mapped in the mQTL6-1 interval that was associated with SDW and GP. The functions of the remaining six candidate genes were associated with energy processes. These genes included an ATP synthase F1 subunit alpha gene (327195227) that mapped in the mQTL3-2 interval associated with GE and GP; a glyceraldehyde 3-P dehydrogenase gene (At3g04120) that mapped in the mQTL4-2 interval associated with GE and RDW; a V-type (H+)-ATPase domain gene (326509331) that mapped in the mQTL4-3 interval associated with GE, SDW, and RDW; a phosphoglucomutase gene (At1g70730) that mapped in the mQTL6-1 interval associated with SDW and GP; a 3-phosphoglycerate kinase gene (226247007) that mapped in the mQTL6-2 interval associated with GE, GP, SDW, and RDW; and an isocitrate lyase gene (AT3G21720) that mapped in the mQTL7-2 interval associated with GP and RDW. These key proteins are responsible for glycolysis during seed aging, which plays a major role in the maintenance of the intracellular redox state and seed vigor [11], [52]. Our results showed that seeds were subject to oxidative stress during artificial aging treatments and mounted a protective response through modification of the glycolytic pathway [9], [39], [50], [51].

### Protein Metabolism is Major a Factor in Seed Vigor during Artificial Seed Aging

Previous studies showed that that protein metabolism plays an important role in seed germination during artificial seed aging and that several metabolic functions are affected, including protein folding, protein translocation, thermotolerance, oligomeric assembly, and switching between active and inactive protein conformations [8], [53]. Rajjou and Debeaujon [8] demonstrated that simultaneous impairment of these functions was closely linked to the loss of seed vigor and Prieto-Dapena et al. [10] showed that transgenic seeds overexpressing a heat stress transcription factor exhibited increased accumulation of HSPs, resulting in improved resistance to artificial aging treatments in seeds. The preservation of a robust stress response and protein turnover mediated by HSPs is a requirement for all organisms [54].

Eight candidate genes identified within mQTLs identified in this study were functionally associated with protein metabolism. Four of the candidate genes had functions related to general protein metabolism or translation, including an elongation factor 1-g2 gene (AT1G57720) that mapped in the mQTL4-3 interval associated with GE, SDW, and RDW; an elongation factor 1B-g gene (AT1G09640) that mapped in the mQTL6-1 interval associated with SDW and GP; a calreticulin 1 gene (AT1G56340) that mapped in the mQTL7-1 interval associated with GE and RDW; and an Asp aminotransferase gene (At5g19550) that mapped in the mQTL3-3 interval associated with SDW and RDW. The other four candidate genes encoded HSPs including a hypothetical ACD ScHsp26-like gene (242056533), an HSP16.9 gene (195605946), an HSP17.2 gene (162459222) that mapped in the mQTL3-1 interval associated with GE and GP, and an hsp20/alpha crystallin family protein gene (At5g51440) that mapped in the mQTL3-4 interval associated with GE and GP. The proteins with chaperone activities encoded by the candidate genes (Hsp26, hsp20, HSP16.9, and HSP17.2) are favored targets for oxidation, presumably because they act as shields to protect other proteins against ROS damage [55]. Other chaperone proteins such as calreticulin (AT1G56340) were also oxidized in deteriorated dry seeds [8].

In addition, five candidate genes identified within mQTLs identified in this study were associated with protein modification and signal transduction functions. These genes included a ubiquitin E2 gene (At1g45050) involved in protein modification and a calcium-dependent protein kinase (CaMK) gene (302810918) involved in signal transduction, both of which mapped in the mQTL3-2 interval associated with GE and GP; a gene containing a predicted RING-finger domain (224143836) involved in protein targeting and storage that mapped in the mQTL3-3 interval associated with SDW and RDW; a CAAX prenyl protease 1 gene (226508796) with hydrolase activity that mapped in the mQTL4-2 interval associated with GE and RDW; and a cucumisin-like Ser protease (At5g67360) that mapped in the mQTL5-2 interval associated with GP. The RING-finger domain-containing protein (224143836) and the protein-degrading ubiquitin E2 protein (At1g45050) play key roles in the ubiquitination pathway [56]. CaMK (302810918) is active in protein phosphorylation through the transfer of phosphate from ATP to protein substrates. Protein phosphorylation plays a key role in many signaling pathways such those involved in responses to stress caused by cold, heat shock, desiccation, peroxide, salt, or ABA [48], [49]. The regulation of CaMK in maize seeds subjected to artificial aging might alter signal transduction.

### Other Pathways Involved in Seed Vigor during Artificial Seed Aging

Embryo cells undergo active division and expansion during seed germination [57]. Seeds germinate much more slowly after artificial aging treatments [58], suggesting that these processes related to cell growth might be affected. Two candidate genes had functions related to cell growth and division. A predicted cyclin-dependent kinase (CDK) A gene (224061823) mapped in the mQTL1-4 interval associated with RDW and GE and a MEK homolog1 gene (162459414) mapped in the mQTL3-3 interval associated with SDW and RDW. In addition, a lipoprotein gene (195658029) involved in lipid metabolism mapped in the mQTL4-2 interval associated with GE and RDW. CDK and MEK homolog1 play pivotal roles in the regulation of the eukaryotic cell cycle. For example, inappropriate activation of CDK can lead to apoptosis [59], [60].

In conclusion, the QTLs and the candidate genes identified in this study provide valuable information for identifying additional quantitative trait genes. The alleles for seed vigor could be useful targets for MAS to produce germplasm with improved resistance to artificial aging treatments.
